# Two-Dimensional Pnictogen for Field-Effect Transistors

**DOI:** 10.34133/2019/1046329

**Published:** 2019-10-16

**Authors:** Wenhan Zhou, Jiayi Chen, Pengxiang Bai, Shiying Guo, Shengli Zhang, Xiufeng Song, Li Tao, Haibo Zeng

**Affiliations:** ^1^Key Laboratory of Advanced Display Materials and Devices, Ministry of Industry and Information Technology, College of Material Science and Engineering, Nanjing University of Science and Technology, Nanjing 210094, China; ^2^Jiangsu Key Laboratory of Advanced Metallic Materials, School of Materials Science and Engineering, Southeast University, Nanjing 211189, China

## Abstract

Two-dimensional (2D) layered materials hold great promise for various future electronic and optoelectronic devices that traditional semiconductors cannot afford. 2D pnictogen, group-VA atomic sheet (including phosphorene, arsenene, antimonene, and bismuthene) is believed to be a competitive candidate for next-generation logic devices. This is due to their intriguing physical and chemical properties, such as tunable midrange bandgap and controllable stability. Since the first black phosphorus field-effect transistor (FET) demo in 2014, there has been abundant exciting research advancement on the fundamental properties, preparation methods, and related electronic applications of 2D pnictogen. Herein, we review the recent progress in both material and device aspects of 2D pnictogen FETs. This includes a brief survey on the crystal structure, electronic properties and synthesis, or growth experiments. With more device orientation, this review emphasizes experimental fabrication, performance enhancing approaches, and configuration engineering of 2D pnictogen FETs. At the end, this review outlines current challenges and prospects for 2D pnictogen FETs as a potential platform for novel nanoelectronics.

## 1. Introduction

While Moore's law is approaching its limit, research and development on new materials, novel device integration, and innovative architecture are on-demand to sustain and extend the microprocessor revolution. As a promising new material family, two-dimensional (2D) materials could lead novel devices with new functions never affordable by mainstream semiconductors nowadays.

To meet the essential requirement, field-effect transistors (FETs), for potential functional devices, numerous 2D materials have been explored, covering a wide range from semimetals, semiconductors to insulators, such as graphene [1], silicene [2, 3], black phosphorus [4, 5], tellurene [6, 7], transition metal dichalcogenides [8, 9], hexagonal boron nitride [10, 11], and many others [12]. Different from the conventional bulk configuration, atomic-thin 2D semiconductor materials are insensitive to short channel effects, which present the scaling potentials for sub-10 nm gate length. Besides, a widely dispersing bandgap and carrier mobility of 2D materials could meet the requirement from low power consumption to high speed. With the successful demonstration of black phosphorus FET in 2014, there has been a widespread device research attention to emerging 2D-layered pnictogen, namely, phosphorene, arsenene [13], antimonene [14], and bismuthene [15, 16]. In the past five years, thousands of articles about 2D pnictogen materials have been reported, both in theory and experiment, covering the range from synthesis to applications [17].

This review will focus on 2D pnictogen materials and their integration into FETs.  Section 2 will introduce the crystal configuration and electronic properties of 2D pnictogen, followed by a brief description of the latest experimental progress of 2D pnictogen in Section 3.  Section 4 will provide a detailed summary on material properties and channel dimension effects to the device behavior of 2D pnictogen FETs. Moreover, Section 4 will discuss the device design for high-performance FETs in terms of the heterostructure, functionalization, contact, gate, and dielectric engineering. Finally, Section 5 will summarize the current research progress, challenges, and outlook on the development of 2D pnictogen FETs.

## 2. Crystal Configuration and Electronic Band Structure

### 2.1. Crystal Configuration

Layered pnictogen materials, possessing a series of typical allotropes, present various crystal structures. Expressly, phosphorus exists in several common allotropes, including white, red, violet, and black forms [18]. Among these allotropes, bulk black phosphorus is the most thermodynamically stable configuration under ambient conditions, which crystallizes in layered orthorhombic structure (*α* phase), forming parallel puckered atomic layers with space group *Cmca* by van der Waals interactions. Bulk black phosphorus demonstrates a typical semiconducting property with a bandgap of 0.3 eV and carrier mobility of about 10^3^ cm^2^ V^−1^ s^−1^ [19, 20]. Under a certain pressure, black phosphorus transforms into a semimetallic *β* phase with space group *R*$\bar{3}$*m* [21]. The *β* phase shows a layered rhombohedral structure, holding a double-atom layer consisting of many interlocked, ruffled six-membered rings.

Metallic arsenic possesses three common allotropes named with gray, yellow, and black arsenic. As the most stable form with a layered rhombohedral structure, gray arsenic with a *β* phase exists in nature and has been known for more than a thousand years [22]. The metallic electronic structure of gray arsenic leads to the partial overlap of bands near the *T* and *L* points in a reciprocal space [23]. When heated to 370 K, arsenic presents phase transition from rhombohedral to orthorhombic structure, which is similar to the bulk black phosphorous structure [24]. Similarly, antimony also has three known allotropes under normal conditions, which are gray, black, and explosive antimony. Layered gray antimony is the most stable phase and presents the same rhombohedral structure as gray arsenic. Both bulk gray arsenic and antimony reveal typical semimetal characteristics. Interestingly, a rhombohedral structure is the only stable form of bulk metallic bismuth, which is also a naturally layered structure like gray arsenic and antimony.

**Figure 1:**
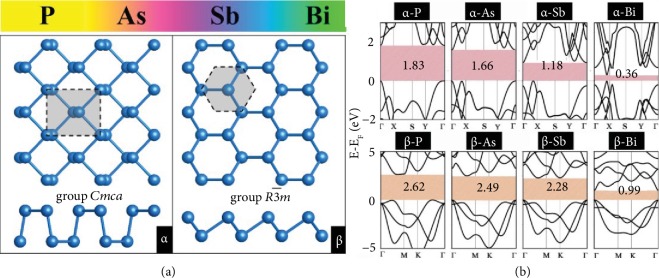
(a) Structures of P, As, Sb, and Bi monolayers for *α* and *β* phases. (b) Electronic band structures of pnictogen monolayers at the HSE level. Reproduced from Ref. [14] with permission from Wiley, copyright 2016.

The 2D pnictogen materials stemmed from phosphorus, arsenic, antimony, and bismuth are called phosphorene, arsenene, antimonene, and bismuthene. Through density functional theory, many allotropes of phosphorene with compacted honeycomb or nonhoneycomb nanostructures have been predicted and investigated systematically [25–27]. Among these allotropes, the puckered (*α*-phosphorene) and buckled (*β*-phosphorene) monolayers are the most common structures (Figure 1(a)), corresponding to the monolayer structures of black and blue phosphorus crystals. The puckered form is the most stable allotrope for phosphorene, whereas the buckled arsenene, antimonene, and bismuthene are the most thermodynamically stable due to the lowest binding energy in all allotropes [14].

### 2.2. Electronic Band Structure

Much theoretical research has been performed to investigate the bandgap and electronic structures of 2D pnictogen materials to explore their electronic and optoelectronic properties. Figure 1(b) presents the bandgaps of *α* and *β* phase phosphorene, arsenene, antimonene, and bismuthene based on the Heyd-Scuseria-Ernzerhof (HSE) hybrid method [28], which is more credible compared with the Perdew, Burke, and Ernzerhof (PBE) method [22].

Black phosphorene can maintain a direct bandgap when thinned to few layer even monolayer, while the bandgap values are dependent on layer number [29]. Based on Green's function and screened Coulomb Interaction (GW) method [30, 31], it reveals that a tunable bandgap of black phosphorous increases from 0.3 to 2.0 eV with the thickness decreasing from bulk to monolayer. By contrast, semiconducting blue phosphorene exhibits a much larger indirect band gap of 2.62 eV [25]. As the most stable allotropes, buckled arsenene and antimonene monolayers hold 2.49 and 2.28 eV indirect bandgap at the HSE level, while puckered ones have a smaller bandgap of 1.83 and 1.66 eV, respectively. Due to heavy element that induces a strong spin-orbit coupling (SOC) effect, the bandgap of *β*-antimonene with SOC fixes to 1.55 eV based on the HSE level. As the last one of 2D pnictogen materials, semimetallic-layered *β*-bismuthene crystal is characterized by a small density of states around the Fermi level, while its monolayer reveals a narrow gap (0.99 eV based on HSE) for band structures [15, 32, 33]. Significantly, as the heaviest element in pnictogen, a strong SOC effect should impact the band structure of bismuthene, and the fundamental bandgap decreases to 0.32 eV along with an electronic transition from direct to indirect.

## 3. Material Preparation

### 3.1. Mechanical Exfoliation

**Figure 2:**
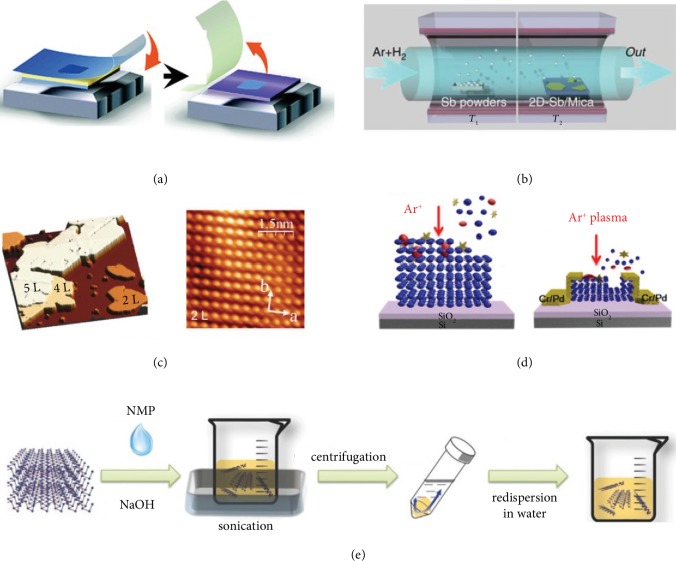
(a) Schematic diagram of the mechanical exfoliation process. Reproduced from Ref. [64] with permission from the Royal Society of Chemistry, copyright 2018. (b) Illustration of the sample synthesis configurations. Reproduced from Ref. [43] with permission from Nature Publishing Group, copyright 2016. (c) STM topographic image of few-layered *α*-antimonene. Reproduced from Ref. [50] with permission from Wiley, copyright 2019. (d) Schematic illustration of the plasma treatment for black phosphorus nanosheets. Reproduced from Ref. [53] with permission from the American Chemical Society, copyright 2015. (e) Schematic diagram of the fabrication process of liquid phase exfoliation. Reproduced from Ref. [65] with permission from Wiley, copyright 2015.

As the first method to obtain graphene, mechanical exfoliation has been developed for decades. It is widely used in the preparation of high-quality black phosphorene, because of simplicity and portability [8, 34, 35]. Phosphorene can be exfoliated from bulk phosphorus crystals and transferred via a scotch tape (Figure 2(a)) onto commonly used device substrates such as SiO_2_/Si, glass, and SiN. Afterwards, a protective capping layer is often used to avoid severe degeneration for phosphorene under ambient conditions, for example, AlO*_x_* [36], PMMA (poly-methyl methacrylate) [37], PMMA/MMA (methyl methacrylate) [38], PMMA/PVA (polyvinyl alcohol) [34], PDMS (polydimethylsiloxane) [39], and ZEP520A [40]. In 2016, antimonene was prepared by micromechanical exfoliation successfully [41]. Nevertheless, the typical mechanical exfoliation is hard to fabricate monolayer antimonene and needs to be improved due to its stronger interlayer van der Waals interaction. Ares et al. achieved thick flakes and a little number of few-layer nanosheets through replacing the scotch tape with a viscoelastic polymer [41]. The soft viscoelastic polymer promotes a higher yield of nanosheets on the polymer surface. By pressing the polymer against a SiO_2_ substrate, thin antimonene nanosheets are obtained controllably with a large area. Also, the stable monolayer *α*-arsenene exfoliated from the natural mineral by Chen et al. and Zhong et al. shows more competitive in-plane anisotropies than any other known 2D crystals [42]. Although mechanical exfoliation confirms the existence of antimonene, large quantity synthesis through this procedure is complicated.

### 3.2. van der Waals Epitaxy

This technique employs classical epitaxy growth of material on the substrate to prevent dangling bond formation on its surface. The epitaxial layers contact with the substrate by van der Waals forces rather than chemical bonding. Ji et al. have grown high-quality few-layer *β*-phase antimonene on a variety of substrates [43]. The experimental procedure (Figure 2(b)) adopts a two-zone tube furnace with separate temperature controllers. Antimony powder is put in the *T*_1_ area and heated up to 660°C to produce antimony vapor, while the substrates are put in the *T*_2_ area of the furnace under 380°C that produces antimony condensation. Using Ar/H_2_ gas as transporting flow, antimony vapor is transferred from the *T*_1_ to the *T*_2_ zone causing a soft-landing and diffusion of Sb atoms on the substrate and then allowing crystallization. Mainly, mica is a suitable substrate for van der Waals epitaxial growth of few-layer antimonene because of its ultrasmooth surface without dangling bonds.

### 3.3. Molecular Beam Epitaxy

Epitaxial synthesis is a scalable means to fabricate the quantity of crystalline 2D materials [44–48]. Epitaxial growth of monolayer *β*-antimonene on the PdTe_2_ substrate has been reported by Wu et al. [49], which is the most air-stable phase. Recently, by the van der Waals heteroepitaxial growth method, Shi et al. have grown monolayer *α*-antimonene successfully on the WTe_2_ substrate with the controllable thickness (Figure 2(c)) [50]. The ultrastable *α*-antimonene demonstrates a hole-doped character with high electrical conductivity and linear band dispersion around the Fermi level. Besides, Reis et al. have grown monolayer bismuthene by epitaxial deposition on a SiC (0001) substrate. Importantly, they obtained a bandgap of 0.8 eV and the existence of conductive edge states by scanning tunneling spectroscopy, which is consistent with theoretical results [51].

### 3.4. Plasma-Assisted Fabrication

The first successful experiment to fabricate stable monolayer phosphorene is through mechanical exfoliation and a subsequent Ar^+^ plasma thinning process [52]. This strategy is effectively controllable for the fabrication of few-layer phosphorene with thickness control based on modulated plasma treatment (Figure 2(d)) [53]. Also, Tsai et al. have synthesized multilayer arsenene successfully on the InAs substrate by the plasma-assisted process [54].

### 3.5. Liquid Phase Exfoliation

Considering the low yield of mechanical exfoliation, researchers proposed a new method, liquid phase exfoliation (Figure 2(e)), to prepare 2D materials with success in high-yield and large-scale production of phosphorene, antimonene, and bismuthene. Brent et al. firstly produced phosphorene in *N*-methyl-2-pyrrolidone (NMP) utilizing liquid phase exfoliation in 2014 [55]. Then, Gibaja et al. reported few-layer antimonene flakes with high quality via liquid phase exfoliation [56]. Recently, assisted by sonication and without any additional surfactant, a liquid phase exfoliation procedure to fabricate few-layer arsenene is reported by Beladi-Mousavi et al. [57]. Besides, Huang et al. successfully produced monolayer bismuthene nanosheets through ice-bath sonication assisted with liquid phase exfoliation [58]. Liquid phase exfoliation is a powerful method with the potential to be scalable for mass fabrication of layers suspended in a variety of solvents.

Other methods to synthesize few-layer pnictogen are also available, for example, pulsed laser deposition [59], electrochemical exfoliation [60], solvothermal synthesis [61], and aqueous shear exfoliation [62]. Pulsed laser deposition is an optional approach to obtain 2D monolayer materials. Depending on this method, Yang et al. have successfully deposited wafer-scale black phosphorus nanosheets at relatively low temperatures [59]. Notably, through the same approach, Yang et al. recently reported the direct growth of bismuthene nanosheets in similar scale and property again, obtaining a thickness-dependent bandgap (0.075-0.2 eV) by characterization of optical properties [63].

## 4. Integration and Characterization of 2D Pnictogen FETs

As the indispensable fundamental unit of modern integrated circuits, FET is probably the most widely studied semiconductor device. Engaging 2D materials in FET could improve its performances effectively due to the absence of dangling bonds, undesirable coupling with phonons, and creation of interface states. Moreover, the 2D channel structure is theoretically robust to against short channel effects, making it possible to continuously prolong Moore's law below 10 nm [66]. As a new family of nanomaterials, 2D pnictogen inherits the common properties including high elasticity and superior flexibility [67], making it suitable for flexible electronics.

Their tunable bandgap and competitive mobility meet the requirement for high-performance power gain, analog amplifiers, and circuits, which promote a higher on/off ratio and faster operation [68]. Therefore, 2D pnictogen (e.g., black phosphorus) transistors exhibit a higher on-current and faster speed compared with TMD-based FETs. Due to the direct bandgap structure of black phosphorus, the bandgap could be deduced from an absorption spectrum, ranging from visible light to infrared. This feature leads to better optoelectronic applications for black phosphorus with respect to monolayer TMD materials [69]. In addition, after structural optimization by encapsulating, the device performance of 2D pnictogen could experience a substantial improvement approaching that of Bi_2_O_2_Se, a novel 2D semiconducting material, presenting significant potential for next-generation electronic applications [70]. Therefore, FETs based on 2D pnictogen hold great promise for future flexible electronics and inherited compatibility with other 2D materials due to van der Waals interaction among the layered structures.

### 4.1. Device Behaviors

**Figure 3:**
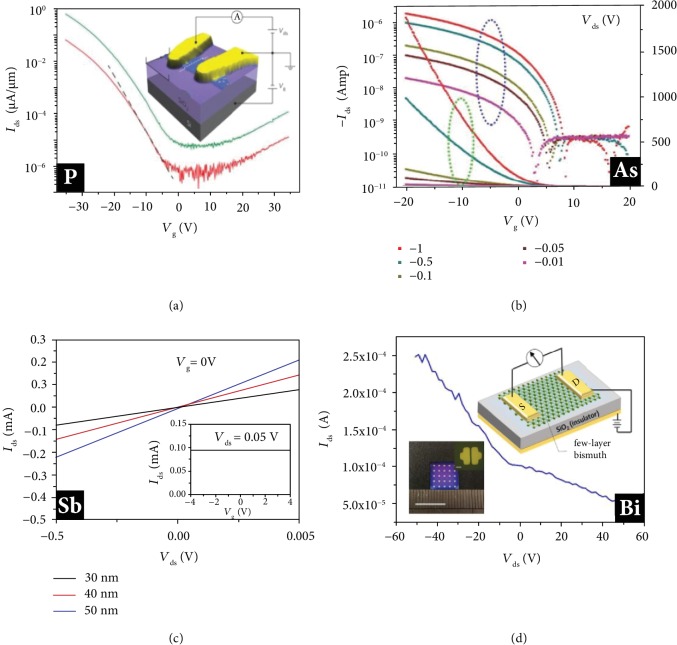
Phosphorene, arsenene, antimonene, and bismuthene FETs. (a) Schematic of the device structure of a few-layer phosphorene FET. Reproduced from Ref. [8] with permission from Nature Publishing Group, copyright 2014. (b) Transfer characteristics of the monolayer arsenene device. Reproduced from Ref. [74] with permission from Wiley, copyright 2018. (c) Output characteristics of antimonene devices. Reproduced from Ref. [43] with permission from the American Chemical Society, copyright 2015. (d) Transfer curve of the FETs based on 10 nm thick bismuthene flack. Reproduced from Ref. [63] with permission from Wiley, copyright 2019.

In 2014, the FETs based on few-layer black phosphorus had been successfully fabricated [8, 9]. As a schematic illustration of the device structure presented in Figure 3(a), black phosphorus nanosheets prepared by mechanical exfoliation are transferred onto doped silicon wafers with thermally grown silicon dioxide as gate dielectrics. Typical black phosphorus FETs hold significant drain current modulation (~10^5^) and thickness-dependent field-effect mobility (~1000 cm^2^ V^−1^ s^−1^) at room temperature [8, 9, 71]. Liu et al. reported that direction-dependent on-state current and switching speed are highly correlated to the carrier effective mass in the corresponding transport direction [72]. Comparing with MoS_2_ FETs, phosphorene FETs possess a faster switching speed and generate higher current density.

Soon after, black arsenic-phosphorus (AsP) was synthesized with highly tunable chemical compositions and fabricated as back-gate FETs [73]. Different transport measurements reveal the semiconducting nature of black AsP materials and their potential applications in logic electronics. In 2018, Zhong et al. reported FETs based on stable black arsenene crystals, presenting high carrier mobility and large *I*_on_/*I*_off_ ratios (Figure 3(b)) [74]. In another study, Ji et al. fabricated transistors by utilizing antimonene polygons with HfO_2_ as a top-gate dielectric, whose channel materials were grown by van der Waals epitaxy (Figure 3(c)) [43]. They pointed out that the competitive conductivity of antimonene combined with the outstanding optical transparency might attract interesting optoelectronic applications. Recently, centimeter-scale bismuth nanosheet-based FETs with considerable carrier mobility have been reported by Yang et al. (Figure 3(d)) [63]. So far, all 2D pnictogen entities, except nitrogen, have been experimentally integrated into FETs, demonstrating the excellent feasibility and great potential in electronics and optoelectronics.

#### 4.1.1. Ambipolar

Channel materials with ambipolar property could present symmetric n- and p-type behavior in charge transport. It is often desired to realize simplified and space-saving circuit designs. Das et al. reported enhanced electron and hole transport character in phosphorene FETs with field-effect mobility of 172 and 38 cm^2^ V^−1^ s^−1^ for holes and electrons, respectively [75]. Zhu et al. introduced an ambipolar phosphorus amplifier with voltage gains of ~8.7, achieved at symmetric DC bias of *V*_GS_ = −1.6 V and *V*_DS_ = −2.1 V with source or gate served as input terminal and drain served as output terminal [68]. Although charge trapping sites exist on the surface, black phosphorus FETs are still able to demonstrate intrinsic ambipolar characteristics [76, 77]. Similarly, black arsenene FET demonstrated ambipolar charge transport behavior, which reveals higher or comparable electronic [42], thermal, and electric transport anisotropies between the armchair and zigzag directions than any other known 2D crystals.

#### 4.1.2. Anisotropy

**Figure 4:**
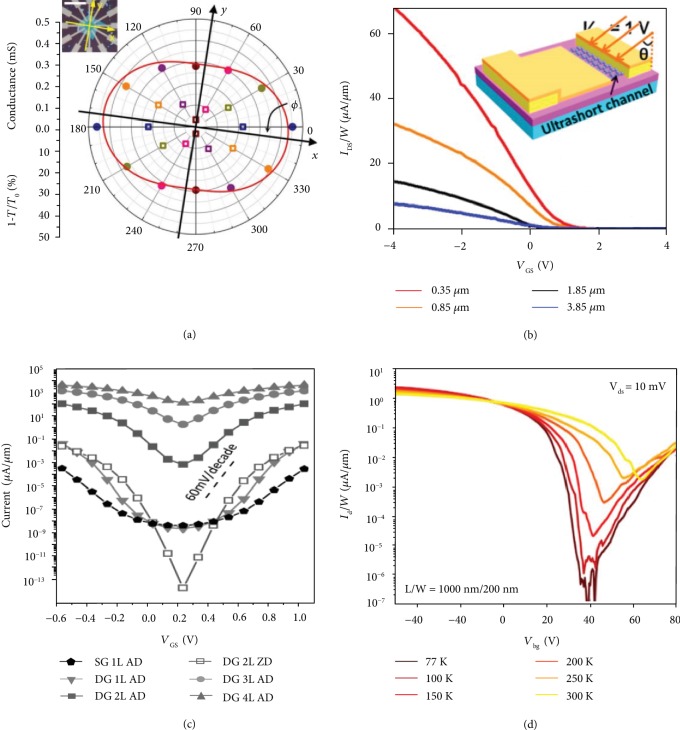
(a) DC conductivity and IR relative extinction measured along with the same six directions on this black phosphorus flake and plotted in polar coordinates. Reproduced from Ref. [81] with permission from Nature Publishing Group, copyright 2014. (b) Transfer characteristics of the top-gated black phosphorus FETs. Reproduced from Ref. [82] with permission from the American Chemical Society, copyright 2015. (c) *I*_D_‐*V*_G_ curves for black phosphorene FETs with 10 nm gate length at *V*_D_ = 0.5 V. Reproduced from Ref. [72] with permission from the American Institute of Physics, copyright 2015. (d) Gate sweep of *I*_d_‐*V*_bg_ curves for the device with *L* = 1000 nm starts with negative voltage. Reproduced from Ref. [83] with permission from the American Chemical Society, copyright 2019.

Inspired by a few preliminary studies, the anisotropy effects and the unique properties of pnictogen on the device performances have been extensively investigated in experiment and theory [59, 78, 79]. For instance, anisotropic photocurrent generation in black phosphorus FETs has been investigated through resolved polarization-dependent photocurrent characterization [80]. Because of the crystal orientation-dependent absorption in black phosphorus, anisotropic photocurrent response is observed near the black phosphorus-electrode contact area in its FET devices. According to the polarization-resolved infrared spectroscopy and angle-resolved DC conductivity, black phosphorus demonstrates large and anisotropic in-plane optical conductivity (Figure 4(a)) [81].

### 4.2. Device Parameters/Optimization

In many FET applications, it is desired to reduce the subthreshold swing and increase on-state current and obtain well-defined saturation of the drain current in the high drain-source bias region [75, 84–90]. Thus, it is necessary to optimize device parameters such as channel dimensions and contact resistance.

#### 4.2.1. Channel Length

Miao et al. fabricated top-gated black phosphorus FETs combining electron beam lithography with angle deposition, which presented high performance (Figure 4(b)). The channel length of the FETs can be reproducibly modulated in the range from 20 to 70 nm through the evaporation angle control [82]. With the advancement of device fabrication technology, a large number of FETs with short channel length were fabricated successfully, including 5 nm carbon nanotube FET [91], sub 10 nm even to 1 nm channel-length MoS_2_ FET [92–94], and silicon-based MOSFETs. Benefited by configuration properties of 2D black phosphorus, the transistors revealed relatively weak short channel effects. Meanwhile, a comparable *I*_on_/*I*_off_ ratio of 10^2^ is obtained even with a 20 nm channel. Lam et al. reported the sub-10 nm black phosphorene with double gate field-effect transistors. The transistors exhibit 50 fs intrinsic delay and 10^4^*I*_on_/*I*_off_ ratio based on the nonequilibrium Green's function formalism and Ballistic device model [95–97]. The various performance indexes of the optimal sub-10 nm phosphorene FETs expressively meet the requirements of ITRS for high-performance devices [86–88]. Through selecting the appropriate transport direction, *I*_on_/*I*_off_ even can be boosted more than 10^8^ induced by enhanced tunneling efficiency [98]. Besides, Wang et al. systemically investigated the relation between device performance and a channel length of arsenene and antimonene FETs based on ab initio methods [99]. The simulated MOSFETs with sub-10 nm or even 4 nm channel exhibit outstanding electrical performances and can meet both the low power and high-performance application requirements in the ITRS [85].

#### 4.2.2. Channel Thickness

The thickness of 2D semiconductors is a significant parameter to determine its fundamental electronic properties and device performances. Since the bandgap of black phosphorus is a function of layer numbers, thickness controlling is a feasible and efficient approach to control the performance of FETs (Figure 4(c)) [89, 100–102]. Das et al. reported the current density and *I*_on_/*I*_off_ ratio of phosphorene FETs emerging to be impacted (~10^2^) by the layer thickness, which is confirmed from the transfer characteristics by a robust technique [103]. In theory, Yin and Yoon suggested that monolayer black phosphorus FETs could yield comparable on-state current with bulk phosphorus while without presenting a shortcoming of the lower density of states [104]. Meanwhile, monolayer phosphorene FETs could also maintain steep switching and resist gate-induced drain leakage.

Similarly, the *I*_on_/*I*_off_ ratio of monolayer-trilayer antimonene-based FET also can be boosted up to 4.87 × 10^8^ with 10 nm channel length [105]. Recently, Chang et al. proposed novel antimonene and arsenene tunneling FETs based on the lateral monolayer-multilayer heterostructure [106, 107]. The multilayer area introduces gapless metallic states which prominently enhance the tunneling probability and drain-source current. By ab initio electronic structure and quantum transport computation, even a 1 nm scale multilayer can remarkably boost the current and enable abrupt device switching.

Besides, strain and temperature [77, 108] are also utilized to regulate the fundamental performance of 2D pnictogen FETs. Zhang et al. reported continuous bandgap modulation by mechanical strain applied through flexible black phosphorus FETs, and a sizeable piezoresistive effect was observed in FETs at room temperature [109]. Also, Yan et al. investigated the temperature-dependent transport properties of phosphorene FETs (Figure 4(d)) [83]. In their study, off-state channel current increases with a temperature rising while on-state current decreases, which can be attributed to the charge conduction limiting mechanism.

A much lower off-state channel current leads to lower power consumption, which is critical for digital devices and integrated circuits. The high mobility and gate modulation (*I*_on_/*I*_off_ ratio) make black phosphorus (BP) a suitable material for high-performance logic circuits. Wang et al. first introduced the gigahertz frequency BP FET in 2014 [111]. The device is fabricated with mechanical exfoliated BP, Pd contact pad, and HfO_2_ gate dielectric. A practically operable cut-off frequency, *f*_T_, ~12 GHz is achieved with standard RF characterization including a deembedding process. In 2016, Zhu et al. reported the first bendable gigahertz frequency BP transistors (*f*_T_ up to 7 GHz) on polyimide substrate, making BP the most promising candidate for flexible RF nanoelectronics [110]. Most recently, Te-doped BP [112] or carbide BP [113] FET has been developed to extend the application of BP, for a better combination of high carrier mobility and unchanged gate modulation. According to relevant experimental data shown in Table 1, BP transistor could achieve cut-off frequency exceeding 60 GHz with optimized channel orientation, metal pad, gate dielectric, channel structure, and fabrication process [71, 110–113]. Nevertheless, there is a lack of research in exploring the limit of carrier mobility and on/off ratio for arsenene and bismuthene transistors. Besides, the field-effect characteristic of antimonene has not been reported yet, and further investigation is required.

### 4.3. Heterostructures

Heterostructure, fabricated by stacking different 2D-layered materials together, is a growing trend in device research, as it is an efficient approach to combine the virtues of different 2D layers into one entity. 2D pnictogen consists of covalently bonded, dangling-bond-free lattice and is weakly connected to neighboring layers by van der Waals interactions [114]. Since the layered structure of pnictogen is similar to other 2D-layered structure materials, it is feasible to isolate, mix, and match different atomic layers to fabricate heterostructures and avoid the lattice mismatching and process incompatibility. Hence, 2D pnictogen heterostructures promote a new field for materials engineering and device design to achieve exotic electronic properties.

#### 4.3.1. Black Phosphorus-Graphene

**Figure 5:**
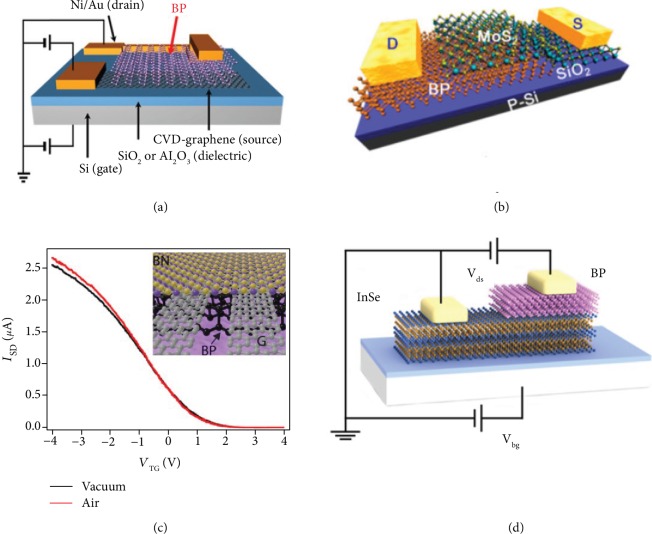
Black phosphorus-based heterostructures FETs. (a) Schematic illustration of the black phosphorus-graphene vertical FET. Reproduced from Ref. [115] with permission from the American Chemical Society, copyright 2016. (b) Schematic of black phosphorus-MoS_2_ heterojunction device. Reproduced from Ref. [122] with permission from the American Chemical Society, copyright 2017. (c) The schematics of the atomically sharp interfaces in encapsulated black phosphorus device. Reproduced from Ref. [127] with permission from the American Chemical Society, copyright 2015. (d) Schematic of the avalanche device of black phosphorus-InSe vertical heterostructures. Reproduced from Ref. [136] with permission from Nature Publishing Group, copyright 2019.

The atomic structure of black phosphorus holds a lone pair of electrons at each P atom, which does not merely delocalize in-plane but also has a substantial effect on out-of-plane atoms. Thus, black phosphorus presents higher out-of-plane conductivities compared to TMDCs, where the chalcogen atoms are insensitive to out-of-plane carrier transport. When considering that out-of-plane transport determining device performance metrics, it is significant to seek a suitable material to combine with black phosphorus as a heterostructure FET. To investigate the charge transport along the out-of-plane direction in black phosphorus, Kang et al. fabricated the vertical FETs based on black phosphorus-graphene van der Waals heterostructures (Figure 5(a)) [115]. The measurement of device characteristics revealed high on-state current densities and *I*_on_/*I*_off_ ratios at low temperature. With high temperature and positive gate voltages, the mechanism of charge transport characteristics is determined by thermionic emission tunneling through the black phosphorus-graphene Schottky barrier. This work revealed black phosphorus as an appealing candidate for van der Waals heterostructure FETs. Besides, through the phosphorus-graphene heterostructure, the interfacial Schottky barrier is tunable due to the weak Fermi level pinning, which is also another significant reason to use graphene as an electrode. We will provide a detailed discussion in Section 4.5.

#### 4.3.2. Black Phosphorus-TMDs

Although many layered materials can be exfoliated to individual atomic planes, not all of them are stable under ambient conditions. Combined with MoS_2_ with high stability, the first black phosphorus-TMD van der Waals heterostructure was fabricated to constitute a p-n diode by Deng et al. [116]. Through illumination measurement under the wavelength of 633 nm, these ultrathin devices exhibit a maximum photodetection responsivity of 418 mA/W and photovoltaic energy conversion of 0.3% external quantum efficiency. Therefore, it is expected to build an electrically tunable device based on the black phosphorus-TMD heterostructure (Figure 5(b)) [117–122]. Through the methods based on cleavage, transfer, alignment, and encapsulation of air-sensitive crystals, Cao et al. reported FETs made from black phosphorus and NbSe_2_ monolayer which is conductive and sufficiently stable under ambient conditions [123]. The approach they provided offers a chance to efficiently expand the range of experimentally accessible 2D crystals and their heterostructures. Significantly, when different 2D materials are stacked together to fabricate van der Waals heterostructure, exotic physics may appear to induce intriguing optoelectronic properties. To understand the critical interlayer coupling in such heterostructures, Yuan et al. systematically investigated the optical and optoelectronic properties of artificial stacks of black phosphorus and MoS_2_/WS_2_ nanosheets. Although phosphorus and MoS_2_/WS_2_ layers both hold a direct bandgap each, the excitons can still be efficiently split by built-in electric fields in black phosphorus-MoS_2_/WS_2_ heterostructures [124].

#### 4.3.3. Black Phosphorus-BN

As a wide bandgap insulator, hexagonal boron nitride (h-BN) holds an atomically flat surface and a natural disorder-free interface contact with other 2D materials, which makes it an excellent candidate for gate dielectric material. Doganov et al. passivated black phosphorus FETs by atomically thin graphene and hexagonal boron nitride (h-BN) and revealed the mechanism of oxidation effect on the physical properties [125]. Gillgren et al. fabricated phosphorene-BN heterostructures by one-dimensional edge contacts. The transport measurements exhibit ambipolar behavior, gate-dependent metal-insulator transition, and considerable mobility with excellent stability in ambient conditions [126]. Stacked graphene/h-BN/MoS_2_ devices have been developed, where MoS_2_ and h-BN were proved to be a valid charge trapping and potential barrier layer, respectively. However, the overall performance is limited by the semimetallic graphene with zero bandgap. Therefore, by replacing graphene with semiconducting black phosphorus, Li et al. fabricated black phosphorus/*h-*BN/MoS_2_ heterostructure FETs showing ambipolar characteristics with prominent *I*_on_/*I*_off_ ratio (10^5^) and remarkable charge carrier mobility (1000 cm^2^ V^−1^ s^−1^). It is anticipated for 2D atomic pnictogen like phosphorene to have rapid sensing of environmental change. Under inert gas conditions, Avsar et al. proposed a fully encapsulated black phosphorus FETs by employing graphene as source-drain electrodes and h-BN as encapsulation layer (Figure 5(c)) [127]. The linear output characteristic curves presented that graphene electrodes result in ohmic contacts to avoid the Schottky barrier limited transport. Chen et al. characterized high intrinsic saturation velocity in black phosphorus FETs as a function of temperature, charge density, and crystalline direction. They determined that the observation of electron-impurity and electron-phonon scatterings results from drift velocity transition point [128].

#### 4.3.4. Black Phosphorus-Other Materials

Beyond graphene, h-BN, and TMDs, several other promising semiconductors have also recently attracted much attention in respect to their prominent properties [110, 129–134]. Jeon et al. utilized the flexible p-type black phosphorus and ZnO to construct van der Waals heterostructures for junction FETs [135]. For these nanodevices with black phosphorus gate switching, high *I*_on_/*I*_off_ ratio (10^4^) and kilohertz dynamic rectification indicate excellent performance and large potential towards future nanoelectronics. Recently, Gao et al. proposed vertical black phosphorus-InSe heterostructures with ballistic avalanche phenomena in submean free path scale (Figure 5(d)) [136]. Their avalanche photodetectors with sensitive midinfrared light detection and steep subthreshold swing exhibit low avalanche threshold, low noise figure, and distinctive density spectral shape.

### 4.4. Doping and Passivation

Air-stability could be a severe issue for 2D pnictogen. It is significant that black phosphorus reacts with O_2_ and H_2_O under ambient exposure over a short period irreversibly to generate phosphoric acid or oxidized phosphorous compounds [137, 138]. Thus, it is imperative to tailor the surface properties and avoid surface degradation of 2D materials [139, 140] via feasible and controllable methods such as doping and passivation. Both approaches could reasonably regulate the electrical transport properties of 2D pnictogen for a better fit into their electronic and optoelectronic applications.

#### 4.4.1. Doping

The approaches to adjust the carrier type of semiconductors can be divided into three categories, which involve substitutional doping, charge transfer, and field-induced doping [141–144]. The conventional doping approach is achieved by incorporating dopants into the host semiconductor lattices, which is largely limited in ultrathin 2D materials. The common dopants include metallic atom, nonmetallic atom, and molecule, as presented in Table 2.

**Figure 6:**
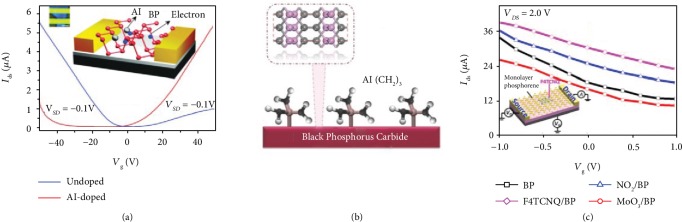
(a) Transfer characteristics of the undoped p-type and the Al-doped n-type black phosphorus FETs. Reproduced from Ref. [147] with permission from Wiley, copyright 2017. (b) A schematic diagram describing the synthesis of phosphorus carbide. Reproduced from Ref. [113] with permission from Wiley, copyright 2019. (c) Transfer characteristic curves of p-type monolayer phosphorene FETs with different adsorbed molecules. Reproduced from Ref. [155] with permission from the American Chemical Society, copyright 2015.

First, alkali metallic atom is often employed as a dopant because of relatively low electron affinities (Figure 6(a)) [145–147]. By the in situ surface functionalization with potassium (K), Han et al. proposed a FET based on giant electron doping of black phosphorene, presenting an ideal device performance with a near-unity ideality factor of 1.007 and around 10^4^*I*_on_/*I*_off_ ratio [148]. Mainly, K modification promotes the electron transport of black phosphorus remarkably. Not only metallic atom but also nonmetallic atom can achieve active surface functionalization [149, 150]. Yang et al. investigated transport performances of black phosphorus FETs doped with tellurium (Te) [112]. The Te doping could dramatically suppress the degradation of black phosphorus, resulting in a high mobility (1850 cm^2^ V^−1^ s^−1^) of FETs at room temperature and pressure. Hence, as a potential route to effectively suppress ambient degradation, a suitable element dopant can promote the development of black phosphorus electronic devices. Besides, combined with a novel carbon doping technique, few-layer black phosphorus carbide FETs fabricated by Tan et al. (Figure 6(b)) demonstrated high hole mobility of 1995 cm^2^ V^−1^ s^−1^ at room temperature [113]. Through DFT calculations, a stable black phosphorus carbide with a lighter effective mass of carriers than its intrinsic ones was obtained. Importantly, its absorption spectrum covers the infrared regime, which is not responded by black phosphorus.

Besides, molecule dopants are also employed to avoid surface degradation of black phosphorus FETs. Based on DFT and NEGF calculations, He et al. theoretically investigated the dynamic transport properties of 2D pnictogen FETs with various molecule adsorption including F4TCNQ, BV, MoO_3_, and gas molecule NO_2_ (Figure 6(c)) [155, 156]. With dynamic transport behaviors, the adsorption of an organic molecule can create additional channels for hole transport, eventually enhancing the performance of p-type FETs. The enormous potential of phosphorene FETs with tunable charge transfer by surface doping is critical for various applications in high-performance devices.

However, surface charge caused by organic molecules may suffer from instability due to complementary semiconductor routes with volatility and incompatibility. For more practical uses, Xu et al. induced Si*_x_*N*_y_* dielectric coating in few-layer black phosphorus as n dopant [154]. They determined that K^+^ centers of Si*_x_*N*_y_* promote active electron doping into BP without external electric field and surprisingly significant improvement of the electron mobility. In most cases, the doping of a proper element is a valid route to suppress the ambient degradation of BP efficiently, and it will accelerate the implementation of BP in novel electronic applications.

#### 4.4.2. Passivation

**Figure 7:**
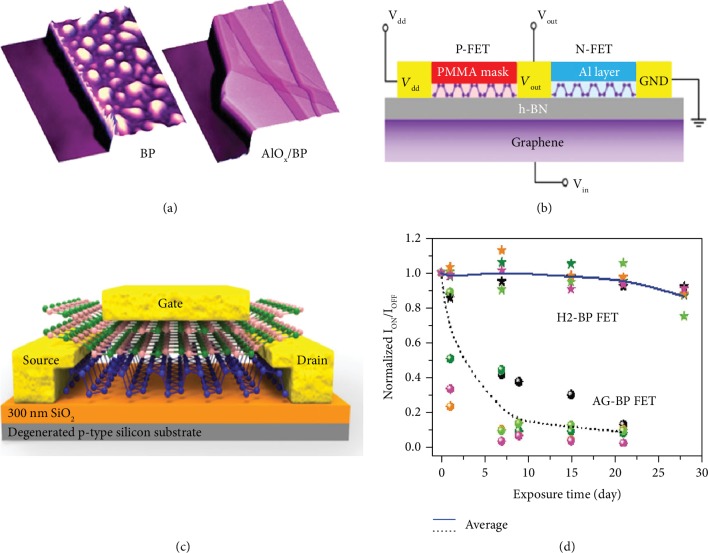
(a) Schematic diagram describing the ambient degradation and AlO*_x_* deposition of black phosphorus. Reproduced from Ref. [36] with permission from the American Chemical Society, copyright 2014. (b) Schematic of the device structure for BP-based logic inverter, which uses BN as dielectric and graphene as a back gate. Reproduced from Ref. [163] with permission from Springer, copyright 2019. (c) Schematic diagram of a local-gated BP FET with h-BN at the top side. Reproduced from Ref. [164] with permission from the American Chemical Society, copyright 2018. (d) Normalized *I*_on_/*I*_off_ ratios by the initial values for AG-BP (dots) and H_2_-BP (star symbols) black phosphorus devices, respectively. Reproduced from Ref. [165] with permission from Wiley, copyright 2018.

Exfoliated 2D phosphorene nanosheets without encapsulation are found to chemically degrade upon exposure to ambient conditions urgently [137]. Passivation is a prominent approach to decrease surface degradation, which not only decreases the surface roughness but also avoids the formation of chemisorbed species [157–162]. Wood et al. investigated how exfoliated black phosphorus degrades to oxygenated phosphorus compounds in the ambient condition through a systematical suite of microscopy and spectroscopy techniques [36]. Their results demonstrate that atomic AlO*_x_* deposition effectively suppresses ambient degradation (Figure 7(a)), leading to relatively high on/off ratios of ∼10^3^ and mobility of ∼100 cm^2^ V^−1^ s^−1^ for over two weeks in ambient environments. Recently, Zheng et al. proposed a feasible and effective electron doping methodology on black phosphorus via in situ surface decoration with Al atom (Figure 7(b)) [163]. After modification by Al atomic layer, the electron mobility of black phosphorus improved mostly by over six times due to the covalent bond at the black phosphorus-Al layer interface, which can also serve as a local gate to modify their transport properties. To enhance the stability and on-current of black phosphorus FETs, Ra et al. investigated the origin of transport limitations of local-gated black phosphorus FETs by comparing the transport performance of h-BN-based device configuration with bottom-gated black phosphorus FETs (Figure 7(c)) [164]. The highest hole mobility of global-gated black phosphorus FETs was 249 cm^2^ V^−1^ s^−1^ with a subthreshold swing of 848 mV/dec, which was maintained entirely for one week without changing the device characteristics in ambient condition.

Moreover, Wan et al. proposed a feasible hydrogen treatment approach to enhance the environmental stability of black phosphorus flakes, which drastically suppressed ambient degradation with little corrosion on the surface even after up to 4 weeks (Figure 7(d)) [165]. Importantly, the performance of FETs still retains over 85% of the initial mobility and *I*_on_/*I*_off_ ratio. Through first-principle calculations, the hydrogen molecules are probably inserted between black phosphorus layers and shift down the conduction band minimum, resulting in reliable surface protection against the formation of superoxide. Besides, Feng et al. passivated the dangling bonds and healed the nanoribbon edge defects effectively in black phosphorus nanoribbons FETs by hydrogenation, promoting nearly hysteresis-free transfer properties [166]. They demonstrate electron mobility of 268 cm^2^ V^−1^ s^−1^, which is comparable to the n-type black phosphorus transistor realized via a doping technique using Al, Cu, or K atoms.

### 4.5. Interfacial Engineering

#### 4.5.1. Contact Resistance

The interface between channel materials and metal electrodes should be carefully treated when fabricating FET devices. The contact resistance turns into a much more dominant factor for device performances in short channel 2D pnictogen FETs. High-quality contact with low contact resistance is desired for practical devices. For traditional channel materials based on bulk semiconductors, substitutional doping and the formation of strong interface bonds are standard methods to establish contacts between electrodes and semiconductors. However, it is difficult for metal electrodes to generate robust covalent bonds on the dangling bond-free surface of 2D materials without deteriorating its electrical property. Several techniques have been employed to realize the fabrication of high-quality and low-resistance contacts, including choosing a metallic electrode, modifying the Schottky barrier height, tuning the interface morphology, and inserting tunneling layers [167–170].

**Figure 8:**
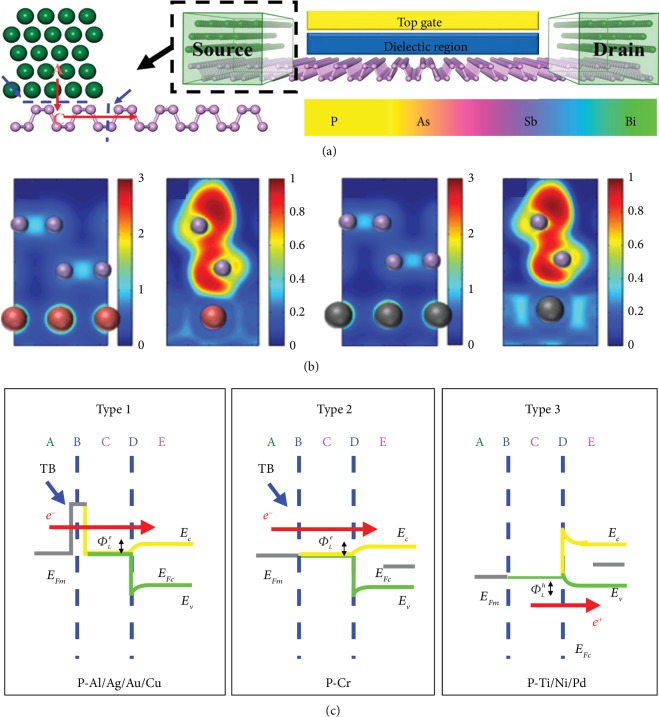
(a) Schematic cross-sectional view of a typical metal contact to intrinsic monolayer phosphorene channel. Reproduced from Ref. [178] with permission from the American Chemical Society, copyright 2016. (b) Electronic structure at the interface of the contact region. Reproduced from Ref. [191] with permission from the American Physical Society, copyright 2014. (c) Three possible band diagrams of the monolayer phosphorene FETs in terms of the quantum transport calculations, depending on the type of metal. Reproduced from Ref. [178] with permission from the American Chemical Society, copyright 2016.

Unlike graphene that can form ohmic contact with metals, 2D pnictogen usually suffers from high contact resistance due to the Schottky barrier at the metal-semiconductor interface, which significantly limits the generation of carrier injection [171–174]. Considering the physical property of Schottky barrier, selecting appropriate metal electrodes for targeted channel materials is fundamentally significant [175–177]. Combining with first-principle calculation and quantum transport simulation, Lu et al. systematically investigated 2D pnictogen FETs in contact with a series of metals with a wide work function range in theory [178–183], including Al, Cu, Ni [184], Sc [185], Ti [186], Py [187], Cr [188], Pd, Er [189], Au, and Ag [190] (Figure 8(a)). In another work, Gong et al. also explored the interfacial properties of the black phosphorus-metal contact, including Cu, Zn, In, TaP, and NbP (Figure 8(b)) [191]. Combining with the results of geometry, bonding structure, density of states, charge transfer, and potential barrier computation, they concluded that black phosphorus-Cu contact is an excellent ohmic contact while the rest metals forming Schottky contacts with black phosphorus. Besides, semimetallic materials are also adopted, such as graphene. According to the results of ab initio quantum transport simulation, the FETs with 2D graphene electrodes present superior performance to those with bulk metal electrodes because of smaller and tunable Schottky barrier heights and the absence of metal-induced gap states in the channels (Figure 8(c)). Notably, the performance limit of 2D pnictogen FETs with graphene electrodes exceeds monolayer MoS_2_, carbon nanotube, and advanced silicon transistors, fulfilling the requirements of the latest ITRS in the next decade.

#### 4.5.2. High-k Dielectric

In short, channel FET devices as the tunneling effect becomes nonnegligible and the leakage current tends to be much higher, resulting from the ultrathin properties of the SiO_2_ dielectric layer. Therefore, high-k dielectric layers are employed to obtain the same gate-insulator capacitance and suppress leakage current in smaller thickness than traditional dielectric materials such as SiO_2_ and BN [192, 193]. Generally, common high-k dielectric materials include Al_2_O_3_, HfO_2_, TiO_2_, HfLaO, and SrTiO_3_.

Liu et al. investigated the few-layer phosphorene FETs behavior with the top gate, which used Al_2_O_3_ as dielectrics by atomic layer deposition [194]. After Al_2_O_3_ capping, a transition of device behavior from p-type to ambipolar conduction has been observed, which is attributed to the alternate of the Schottky barrier heights for electrons and holes. Also, Haratipour et al. presented a thorough investigation of the mobility anisotropy in black phosphorus MOSFETs with HfO_2_ gate dielectrics [195]. The measured room-temperature field-effect mobility was found to be higher in the AC direction, in which the anisotropy ratio was found to increase with carrier concentration in the low-concentration area. Similarly, Liang et al. reported radiation-induced charge trapping and low-frequency noise in passivated black phosphorus FETs with HfO_2_ gate dielectric [196]. The observation of the reduction of ionization induced threshold voltage shift from thinning the gate dielectric. After radiation exposure, field-effect mobility significantly decreased, which could be avoided by reducing the density of O vacancies in the dielectric layer and decreasing the amount of hydrogen in the configuration. Besides, Xiong et al. used HfLaO as the back gate dielectric layer in black phosphorus FETs, which demonstrated excellent device performance [197]. Compared with typical SiO_2_ gate dielectrics, both electronic and optoelectronic properties of these high-k dielectric FETs are greatly enhanced on account of the interface quality improvement.

#### 4.5.3. Ferroelectric Capacitance

**Figure 9:**
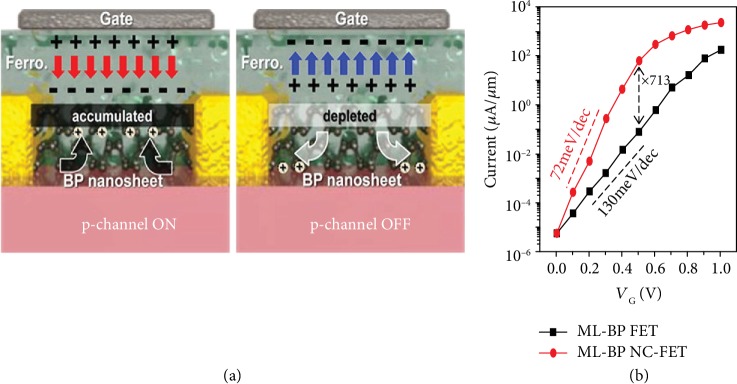
(a) Schematic illustrations of black phosphorus FeFET mechanisms for the program and erase states. Reproduced from Ref. [199] with permission from the American Chemical Society, copyright 2015. (b) Device transfer characteristics of black phosphorus FETs and NC-FETs. Reproduced from Ref. [198] with permission from Nature Publishing Group, copyright 2016.

The significant potential of negative capacitance (NC) is demonstrated to promote FET performance with the steeper slope, which is very useful for voltage/power applications. A negative capacitance FET adopts a thin layer ferroelectric material to the gate oxide of a MOSFET (Figure 9(a)).

Theoretically, black phosphorus has been predicted as a suitable channel material for NC-FETs [198]. Liu et al. investigated the quantum transport properties of NC-FETs based on monolayer phosphorus (Figure 9(b)). In NC-FETs, a ferroelectric (FE) gate layer is adopted and combined with a positive capacitor to obtain a bistable state. The synergetic effect of the polarization in the FE material and the external electric field result in a negative voltage drop through the FE layer, causing a “voltage amplification” that improves the subthreshold characteristics of FETs. Computational results presented that the amplification effect of the ferroelectric layer can be enhanced by black phosphorus. By increasing the thickness of the dielectric, device performance can be further improved, which is embodied in a significantly reduced subthreshold swing of 27 mV·dec^−1^.

By contrast, the relevant experimental progress is still insufficient. Lee et al. reported few-layered black phosphorus FETs with a ferroelectric top-gate insulator [199]. Different from conventional ambipolar FETs, these ferroelectric FETs demonstrate only p-type behavior because of the carbon-fluorine dipole effect of the P(VDF-TrFE) layer. With ferroelectric layer inserting into devices, their experiments presented the highest 1159 cm^2^ V^−1^ s^−1^ mobility and a 10^3^*I*_on_/*I*_off_ ratio. Recently, Tian et al. successfully fabricated NC-FETs based on black phosphorus by a HfZrO ferroelectric capacitor, which exhibits relatively low subthreshold slope with two types of behaviors [200]. The research provides an innovative idea in the exploration of NC-FETs for 2D pnictogen.

### 4.6. Liquid and Dual Gating

**Figure 10:**
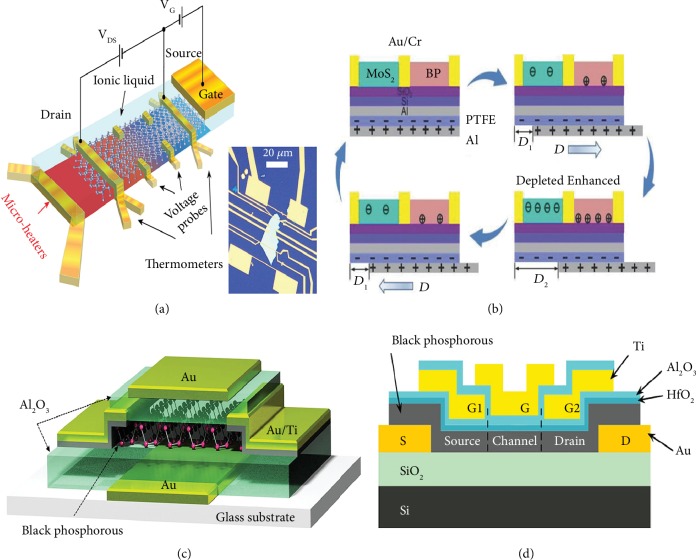
(a) Conceptual image of a black phosphorus-electric double-layer transistor for thermoelectric measurements. Reproduced from Ref. [201] with permission from the American Chemical Society, copyright 2016. (b) The working principle of the tribotronic logic device. Reproduced from Ref. [202] with permission from Wiley, copyright 2018. (c) Schematic 3D view of dual gate black phosphorus FET. Reproduced from Ref. [203] with permission from the American Chemical Society, copyright 2015. (d) Schematic of the black phosphorus reconfigurable electrostatically doped tunneling FET. Reproduced from Ref. [204] with permission from the American Chemical Society, copyright 2019.

Under an external electric field, more charge carriers will accumulate around the channel/electrolyte interface by introducing the different organic and inorganic electrolytes, including polymer electrolytes and ionic liquids or gels. Then, a new kind of transistor named with the electric double layer transistor is designed for examining the charge transport, which is originated from supercapacitors to achieve low voltage operation. Saito et al. reported the electric-double-layer transistor based on the gate tuning of thermoelectric power in black phosphorus nanosheets (Figure 10(a)) [201]. By controlling the thermoelectric power effectively, they revealed that ion-gated black phosphorus possesses a relatively high power at 210 K in the hole-depleted state. The improvement mainly originated from the thinning of the conduction channel analyzed by first principle-based computation. Moreover, Gao et al. reported a tunable tribotronic dual-gate FETs based on black phosphorus and MoS_2_, carrying the sliding mode triboelectric nanogenerator, which could be driven efficiently by triboelectric potential without applying gate voltages (Figure 10(b)) [202]. The tribotronic FETs demonstrate high performance with *I*_on_/*I*_off_ exceeding 10^6^ and off-state current below 1 pA*μ*m^−1^. The creative FET configurations offer active, low-power-consuming, and universal approaches to modulate semiconductor devices and logic circuits based on 2D materials.

In addition, designing dual-gate architecture is also an effective strategy to improve the FET performance. Kim et al. reported dual-gate FETs with black phosphorus channel on a glass substrate, which holds a patterned-gate configuration with Al_2_O_3_ dielectrics on the top and bottom of the channel (Figure 10(c)) [203]. Top, bottom, and dual gate-controlling mobilities were measured to be 92, 277, and 213 cm^2^ V^−1^ s^−1^, respectively. Significantly, these dual-gate FETs presented not only the switching for green and blue OLEDs but also NOR logic functions via separately employing top and bottom input. Recently, Wu et al. reported few-layer black phosphorus TFETs with multiple top gates and electrostatic doping in the source and drain areas (Figure 10(d)) [204]. The devices can be modified to n-type or p-type behavior under electrically tuning the doping types and levels. Moreover, record-high current densities have been obtained in devices due to the suitable choice of materials and cautious construction of device configurations.

## 5. Summary and Perspective

This review summarized the basic structural and fundamental electronic characteristics of 2D pnictogen and its field-effect device configuration design as well as several performance enhancement technologies. 2D pnictogen demonstrates promising physical properties, such as tunable moderate bandgap between graphene and TMDs, holding significant potential for novel device applications with high speed and energy efficient combined. In the device aspect, we discussed recent studies focused on 2D pnictogen transistors featuring high stability and high performance, covering all aspects from their fundamental characteristics to device configurations and integration engineering.

Nevertheless, there are remaining challenges to fulfill the practical use of 2D pnictogen FETs. The first is how to produce large-area 2D pnictogen with good uniformity and high quality, especially for monolayer. Second, there is an urgent concern on the air stability of 2D pnictogen FET devices to keep the performance stable enough for practical applications. Another issue is the interfacial optimization, including dielectrics and contact quality, customized and optimized configuration for 2D pnictogen FET devices.

## Figures and Tables

**Table 1:** Device parameters and properties of 2D pnictogen FETs.

Pnictogen	Bandgap (eV)	Thickness (nm)	Channel L/W (*μ*m/*μ*m)	Mobility (cm^2^/V s)	Gate modulation On/off ratio	Notes	Ref.
Bulk BP	0.3	/	-/-	350 (p); 220 (n)	/	/	[19]
BP	/	18.7	3/15	186.5	10^3^	Pd pad	[71]
BP	/	13	0.5/10.8	233 (p)	10^3^	*f*_T,int_ = 17.5 GHz, *f*_T_ = 7GHz Flexible substrate	[110]
BP	/	10	4.5/2.3	984 (p)	10^5^	/	[8]
BP	/	8.5	0.3/<50	400	2 × 10^3^	*f*_T,int_ = 51 GHz, *f*_T_ = 12GHz	[111]
Te-doped BP	/	7	-/-	1850	1.5 × 10^6^	Te doped	[112]
Carbide BP	/	7	10/5	1995	10^3^	Carbide	[113]
BP	/	~5	1/-	286	10^4^	/	[9]
BP	/	1.9	2/-	172 (p); 38 (n)	2.7 × 10^4^ (p); 4.4 × 10^3^ (n)	/	[75]
As	2.47	/	-/-	59	10^5^	/	[74]
Sb	2.48	/	-/-	/	/	/	[43]
Bi	0.075-0.2	/	30/500	220	<10	/	[63]

**Table 2:** Dopants and properties of 2D pnictogen FETs.

Dopant		Device mobility (cm^2^/V s)	Stability	Ref.
Metallic atom	Li	147	6 weeks	[151]
	K	262 (e)	/	[148]
	Al	1495 (e)	10 days	[145, 146]
	Cu	690 (h)	/	[152]
	MoO_3_	~200 (h)	/	[153]
	Cs_2_Co_3_	27 (e)	/	[153]

Nonmetallic atom	Carbide	1995 (h)	/	[113]
	O	347 (h)	2 weeks	[137]
	S	607	3 weeks	[150]
	Te	818 (h)	3~4 weeks	[112]
	Se	756 (h)	At least 6 days	[149]
	Si*_x_*N*_y_*	956 (h)	1 month	[154]

Molecule	TCNQ	/	/	[155]
	BV	/	/	[156]
	TTF	/	/	[141]
